# ‘You have got to stick to your times’: Care workers and managers’ experiences of working in extra care housing

**DOI:** 10.1111/hsc.12871

**Published:** 2019-10-20

**Authors:** Ailsa Cameron, Eleanor K. Johnson, Simon Evans, Liz Lloyd, Robin Darton, Randall Smith, Jeremy Porteus, Teresa Atkinson

**Affiliations:** ^1^ School for Policy Studies University of Bristol Bristol UK; ^2^ Association for Dementia Studies Institute of Health and Society St Johns Campus University of Worcester Worcester UK; ^3^ Personal Social Services Research Unit University of Kent Canterbury Kent UK; ^4^ Housing Learning and Improvement Network London UK

**Keywords:** care work, extra care housing, older people, social care, the organisation of care

## Abstract

Extra care housing (ECH) has been lauded as an innovative model of housing with care for older people that promotes and supports independent living. The study used a qualitative design to explore how care is delivered in four extra care settings in England over 20 months during 2016–2017. This paper reports findings from semi‐structured interviews with 20 care workers and seven managers. The article argues that, despite being heralded as a new model, care workers in ECH face similar organisational pressures as those working in more conventional settings and, in turn, the care which they are able to provide to residents mimics traditional forms of care.

**What is known about this topic**

- Extra care housing (ECH) is a form of housing with care for older people.
- ECH aspires to support older people to live independently for longer through the flexible provision of care in residents own apartments.
- Similar schemes exist in America and Europe, where they are known as ‘retirement living’, ‘assisted living’ or ‘special housing’.

**What this paper adds**

- The population living in ECH is changing, with more people having complex needs.
- Managers report that these changes are impacting on the way in which care is organised.
- Care workers report that these changes are undermining the ability of ECH to provide responsive and flexible care to residents.

## 1 INTRODUCTION

Improvements in life expectancy over the 20th century, coupled with decreasing fertility rates, have resulted in a substantial ageing of the world's population. Since older people are more likely to experience ill health, any expansion in the number of older people within a population will result in a higher prevalence of disability, disease and chronic conditions (Marengoni et al., 2011). Therefore, while improvements in healthcare continue to support increasing life expectancy, bringing benefits to the economy and wider society, they also put pressure on services such as social care and housing (ONS, 2017; Pynoos, 2018).

Within this context it is no surprise that, in the UK, the provision of housing with care has become a key element of adult social care policy and the focus of a growing body of social care research (Atkinson et al., 2014; Bernard, Bartlam, Sim, & Biggs, 2007; Croucher, Hicks, & Jackson, 2006). While researchers have explored the supply and provision of various models of housing with care, as well as the experiences of older people living in these settings, less attention has been paid to those working in the sector. In beginning to address this gap, this article reports on the experiences of care workers and their managers working in extra care housing (ECH), an increasingly popular form of housing with care.

## 2 WHAT IS EXTRA CARE HOUSING?

In 2003 the Department of Health introduced the ECH Fund to support the development of innovative housing with care options for older people (Department of Health, 2003). While there is no agreed definition of ECH, causing problems for older people and their families making decisions about future care (Verbeek et al., 2017), there is agreement that it is a distinct alternative to other, more traditional, forms of housing and care provision due to three principal characteristics (Evans et al., 2017:20). First, is its focus on ‘supporting independent living in self‐contained accommodation for rent, shared ownership or sale’. Second, ECH departs from other forms of housing with care because it provides access to 24‐hr care on‐site. A significant feature of this care is that it should be responsive and flexible, adapting to the changing needs of older people on a permanent or temporary basis. Finally, a key emphasis of ECH is its provision of communal amenities, such as restaurants, gardens and/or social activities. In addition, residents of ECH have an individual legal right under housing law to occupy. The delivery of care is therefore not part of a tenancy agreement or lease; otherwise registration arrangements would make it a care home. This arrangement is thought to afford residents greater choice and control, and, in terms of care workforce, the ethos is one of ‘to do for’ not ‘to do to’. The development of housing with care is not confined to England. There has been growing interest in this field across America, Australasia and Europe, where it is often referred to as ‘retirement living’, ‘assisted living’ or ‘special housing’ (Howe, Jones, & Tilse, 2013).

Extra care housing preceded many significant social care policy developments in England, including emphases on ‘personalisation’, ‘independence’ and ‘prevention’. It encapsulates these aspirations and marks a move away from traditional models of residential care associated with institutionalisation (Riseborough, Fletcher, & Gillie, 2015). The provision of additional care on a temporary or permanent basis also reflects an aim to ensure services are personalised and respond to the changing needs of individual residents (Evans et al., 2017). The availability of care alongside the provision of communal amenities is thought to support older people to live independently for longer (Evans & Vallelly, 2007). Furthermore, ECH has been identified as a potential solution to problems of social isolation and loneliness (Callaghan, Netten, & Darton, 2009), poor social care outcomes (Bäumker, Netten, & Darton, 2010) and the higher costs associated with residential care (Holland et al., 2015). Researchers have identified that older people living in ECH have high levels of overall satisfaction with their quality of life and social lives (Evans & Vallelly, 2007).

Some research reports less positive experiences, such as a lack of resident satisfaction with care provision (Phillips, Dobbs, Burholt, & Marston, 2015), planned care needs being addressed inappropriately (Wright et al., 2010) and, in particular, loneliness has featured as a significant problem (Burholt, Nash, & Philips, 2013). More recent evidence indicates that the population served by ECH is changing. Skills for Care (2017) suggests that local authority funding for ECH residents is increasingly limited to individuals who have high care needs and that this has an impact upon both existing residents of ECH and the workers who support them.

The research evidence on paid care work suggests that the UK’s social care sector faces a number of challenges, including poor recruitment, low pay, low staffing levels, and high turnover (Hussein, Ismail, & Manthorpe, 2016) and the need for appropriate training of care workers (Gospel, 2015). The literature also suggests an increasing tendency towards the routinisation of care work undermining aspirations for greater flexibility and personalisation of care (Atkinson & Crozier, 2016; Kadri et al., 2018) and a shift in bureaucratic tasks (paperwork) to care workers (Killett et al., 2016). However, since the literature focuses almost exclusively on paid care work in residential, nursing and domiciliary care, little is known about the conditions and experiences of care workers in ECH. Although early evidence suggested a higher ratio of staff hours to residents in ECH compared to other forms of residential care for older people (Callaghan, 2008; Croucher et al., 2006).

## 3 DESIGN AND METHODOLOGY

The Provision of Social Care in Extra Care Housing for Older people (ECHO) study set out to investigate how care is negotiated and delivered in ECH schemes. The study took place in two areas: a unitary authority (area 1) and a county council two‐tier authority (area 2). The areas were chosen to reflect geographical difference and different pressures faced by authorities: urban and increased pressure on land (area 1) and, rural and over provision (area 2). Four ECH schemes were recruited. Sites A and B were based in the unitary authority. Site C, a specialist dementia setting and site D was based in the county council. Table 1 reports the characteristics of the schemes.

**Table 1:** Characteristics of schemes

Area 1 Unitary Authority	Site A	Housing and care provider (not for profit), 54 flats, rent (social landlord), built in 1977, amalgamated with another site in 2007
	Site B	Housing and care provider (not for profit), 49 flats, rent (social landlord), in operation for 12 years
Area 2 County Council	Site C	Separate housing and care providers (care provider is not for profit, housing provider is non‐asset holding, non‐charitable registered society), 42 flats, rent, built in 2015. Specialist dementia scheme
	Site D	Separate housing and care providers (care provider is not for profit, housing provider is non‐asset holding, non‐charitable registered society), 95 flats, available for rent, shared ownership or leasehold sale, majority self‐funders, built in 1998, extended in 2015

Each scheme was visited on four occasions. Semi‐structured interviews were held with residents, care workers and managers of schemes, as well as with commissioners of services (Brinkmann & Kvale, 2015). Residents were interviewed four times across 20 months, managers of schemes and commissioners were interviewed twice, at the beginning and the end of the study, and care workers were interviewed once. An introductory meeting was held with staff and residents at each site prior to fieldwork, to recruit participants. All participants gave written consent prior to the interview. Ethical approval was granted by the Social Care Research Ethics Committee (15/IEC08/0047).

In total, 20 care workers took part in interviews – five from each site – which lasted between 20 and 30 min and took place during or after their shifts. The interviews covered a range of topics including their roles, training and perceptions about ECH. Interviews with managers lasted 60–90 min and explored organisational issues related to how care and support was arranged as well as questions about the policy and practice context. Three of the managers who were interviewed at the beginning of the study left and had been replaced by the time of the second interview, making the final manager sample seven.

All interviews were digitally recorded and transcribed in full. A sample of transcripts was read by one member (AC) of the team in order to develop a coding frame which incorporated a priori codes (for example, related to work pressures) and supplemented with themes arising inductively (for example, unmet need for care). The coding frame was tested and verified by other members of the team (EJ,TA). The full dataset was coded and then organised in relation to emerging themes such as the organisation of care (Spencer, Ritchie, Ormston, O’Connor, & Barnard, 2014).

This paper presents findings from interviews with care workers and their managers. Data are presented using participant codes, for example SACW1 refers to site A, care worker 1. Findings from analysis of the longitudinal data collected from residents are reported elsewhere (Johnson et al., 2019).

## 4 FINDINGS

The findings are presented under five themes which emerged during the analysis and helped explain patterned responses. The themes draw attention to the organisational challenges facing ECH, which appear to be undermining the flexibility and person‐centred objectives at the heart of the model. The themes are as follows: the care worker role; the organisation of care; planning and flexibility; changes in resident mix; and favours.

Before presenting the data, some background information about the sample is provided. All of the managers in this study had previous experience of working in ECH or in traditional forms of care, such as residential care. The length of time that managers had been in post ranged from 6 months to 5 years. For the 20 care workers who took part, this range was greater: six care workers had worked in their current role for less than 12 months, six for between 1 and 5 years, and the remaining eight care workers for at least 5 years, 12 of the 20 had previously worked in the care sector. All participants were women. Despite the similarity in the nature of their work, the role titles of the care workers varied and included care worker, care assistant and care and support worker. There appeared to be little practical distinction between ‘care’ and ‘support’ roles. The care worker sample included one deputy manager and three team leaders who all worked full‐time, while the remaining 16 care workers worked part‐time. Care workers’ contracted hours ranged from 10 to 30 per week. For the purpose of this article, the terms ‘care worker’ and ‘team leader’ are used to refer to those participants who were not managers.

### 4.1 THE CARE WORKER ROLE

When care workers were asked to describe their role, the aims of ECH in relation to supporting people to live as independently as possible, as well as wider policy objectives about choice and control, were implicit in their descriptions. For example, at site B, a team leader who had worked at the scheme for 9 years said:

Basically, the work I feel I do here is we assist vulnerable adults to have more of an independent life, so by doing small tasks for them like helping them to make their food, their personal care, just making their lives a lot easier (SBCW2).

A care worker at site D reported that their role included support tasks, such as escorting to ‘the social club or the lounge or the hairdressers [or] just a few like house bits like packing washing away and just washing‐up’ (SDCW5). For residents with minimal care needs, the role could involve making a ‘welfare visit’. For example, at site A, a care worker described that, for one resident, ‘we don't do any care […] we have a chat with her, make sure she's not fallen in the night’ (SACW5). Such opportunities to talk with residents helped build rapport and were seen as an essential element of the care role for many of the care workers and indeed, these accounts reflect the aspirations of ECH. A team leader at site A, who had previously worked in residential care, suggested that ECH offered an alternative to traditional residential care. She explained:

I like the idea of ECH. Like I said I worked in a care home, didn’t like it, but here […] I like the kind of accommodation, I think it promotes independence, the people can still do quite a lot for themselves […] and they can have fulfilled lives (SACW1).

In contrast, a small number of care workers described their role in terms of a series of tasks to perform and spoke about their work in a more detached and mechanistic manner, reminiscent of traditional forms of residential and domiciliary care (Lee‐Treweek, 1997). At site D, a care worker described her role as:

Getting people up and washed and dressed, showered or bathed in the morning, make breakfast […] Erm obviously put clean clothes on of their choice, whatever they want to wear erm always give them choice and obviously making sure their well‐being is alright (SDCW1).

Similarly detached accounts were offered by care workers at other sites. For example, at site B a participant commented: ‘I do a little bit of everything, personal care, housework, help with their medication. Sometimes I feel like a general dogsbody’ (SBCW1). Independence and choice were sometimes alluded to in these descriptions but echoing findings from research into domiciliary care work (Atkinson & Crozier, 2016), the functionality of the care encounter comes to the foreground.

### 4.2 THE ORGANISATION OF CARE (‘RUNS’)

Across all sites, the daily routines of staff were organised into a series of sequential visits to residents’ apartments which ranged in time from 15 min to 1 hr and 15 min. Care workers from three of the four sites routinely referred to these as ‘runs’, which consisted of each care worker being given a worksheet at the beginning of their shift. This worksheet set out the time at which they were to visit each resident, the nature of the task to perform and the time this should take. Although no participants at site C used the term ‘run’ to describe the organisation of their work, they described a similar system. One care worker told us ‘you come in and you'll pick up your […] rota and it will give you your times and what residents you're going to visit that day’ (SCCW3). Managers at three of the sites also used the term ‘run’ to explain the organisation of care. The manager at site D described how the care needs of residents informed the structure of the staff rota saying ‘We've moved to a completely domiciliary care profile of rostering […] the support that somebody needs is picked up and dropped into a run’ (SDM1).

‘Runs’ were a source of frustration to many, largely because of the focus on a ‘time‐and‐task’ approach (Garwood, 2010). As a care worker at site A noted, ‘You do get stressed sometimes when you've got to rush. You have got to stick to your times’ (SACW2). Staff shortages were an additional pressure that exacerbated the challenges of a time‐and‐task‐based approach. A care worker at site B described the impact of these on her interactions with residents saying, ‘If we're short one member, cause then you have to split a run, which means you have a lot more people […] and you don't have that extra time then to have that little talk with them. You're literally in and out’ (SBCW4).

Many participants wanted to spend more time with residents just talking, they valued these interactions and thought they were beneficial to residents’ well‐being. However, such ‘everyday’ interactions were compromised by the way in which care was organised. A new member of staff at site C said she was struggling to get used to her work, reporting that she found it difficult to stick to her times ‘because I want to spend more time talking to them [residents]’ (SCCW4).

### 4.3 PLANNING AND FLEXIBILITY

Not all residents of ECH have care needs, many move into ECH as a pre‐emptive strategy to maintain independence (Kneale & Smith, 2013). However, all of the schemes reported undertaking initial assessments of care needs before older people moved into the schemes or, when care needs emerged. The study also revealed similarities in how changes in the care needs of residents, a key focus of ECH and often referred to as ‘unplanned care’, were identified and how these changes were responded to by service providers and care workers. Sometimes changes in care needs were raised by residents, or their families, and discussed with care managers. In other cases, changes were necessitated after a period of hospitalisation or a change in health status. However, in most cases, they were identified by care workers who either noted a persistent additional need, such as social isolation, or responded to an immediate need for additional care, for example if a resident had a fall which impeded mobility and self‐care. In these instances, care workers logged ‘unplanned care’, in the paperwork attached to their ‘run’ or they mentioned it to managers. As a care worker at site A described:

‘Normally if it’s additional care, if it’s needed there and then, they’ve taken longer than their time, we have got a worksheet […] which you fill out. The office then sees that and […] it might be a one off that day. But if it’s happening quite regularly then obviously they’ve got to speak to […] their support workers or social workers or what have you say ‘right they actually need extra care because this is not working out now’ (SACW2).

In one‐off cases ‘unplanned care’ was accommodated within the work for that shift. However, if the need arose regularly the resident would be reassessed. If the care needs of self‐funding residents changed, necessitating more care on a regular basis, then a senior carer or the manager would speak to them individually, or would contact their family, to discuss changing the care plan to include the additional care and associated cost.

In 2016, the local authority contract for publicly funded residents in area 1 was amended to cater specifically for unplanned care: 20% of the care provided was expected to meet unpredictable or complex needs, while 40% was allocated to people requiring 5–15 hr of care per week and 40% was allocated to people requiring over 15 hr per week. In area 2 the contract allowed care providers to increase the amount of care for local authority residents for a limited period of up to 3 days and then another 3 days to support a change in care needs before assessment was required. The contractual amendment in area 1 appeared to reflect the increasing complexity of the care needs of residents who were moving into ECH.

Generally, social service departments responded positively to requests for increased funding; however, the speed of response varied, with the consequence that managers had to cover increased care costs until the request was granted with payments often, but not always, back dated. For example, the manager at site A reported:

‘If we have somebody that needs emergency care and they need that care to go in place we will put that care into place. At the same time we will send an email to [name of local authority] to say, this person needs this because of X, Y and Z this is an urgent review and this care needs to start as of today, I need an urgent response’ (SAM2).

This manager said that, in most but not all instances, the local authority would ‘back pay’ the additional costs but her main concern in these situations was the delivery of the care and ‘making sure it's the closest thing to person‐centred and it's around that person not based around a contract’ (SAM2). Similar delays were noted at site B, where the manager reported that she had been waiting over 3 months for the authority to review a case but, in the meantime, the organisation was ‘funding that ourselves […] There's nothing you can do is there? The lady needs the extra care’ (SBM2). Such delays were not confined to area 1. The manager at site C told us of similar delays in back dating funding for changes in care.

### 4.4 CHANGES IN RESIDENT MIX

Reflecting findings from Skills for Care (2017), at two of the sites there was a sense that the needs of those entering ECH, particularly those who were publicly funded, were changing. At site A, the manager reported they were accepting residents with ‘quite complex mental health needs. And exhibiting quite challenging behaviour […] not just to our staff but also to other tenants’. She went on to say ‘my [residents with] complex needs are kind of taking over everything else at the moment, I need to find some balance’ (SAM1). Similarly, a team leader at site B, who had worked there for 17 years, told us:

‘I would say from the time I’ve worked here it’s getting progressively […] higher care if you know what I’m trying to say. Because when I first came to work here it was literally making the meal for somebody and now it’s more of the personal care that’s come in, more manual handling’ (SBCW2).

For sites A and B, which were based in area 1, this increase in care needs might, in part, be explained by changes to the local authority nomination process in 2016, which required that all publicly funded residents were to have care needs of at least 5 hours a week to be eligible to move into ECH. At all sites, we were told that organising staff rotas was difficult when care needs fluctuated significantly and that these difficulties were compounded when new residents moved in with complex needs. This was particularly challenging at site C, the specialist dementia scheme, where the manager said:

‘If you’ve got somebody who’s got a really high need who’s potentially going to need a lot of unplanned care, that’s going to then impact on the care that we’re delivering for the other clients. It’s going to impact on the safety of the scheme and it’s going to impact on the staff’ (SCM1).

Difficulties in managing staffing levels were also experienced when residents with high care needs were admitted to hospital, died or moved elsewhere. For example, the manager at site D told us how several residents with high care needs had recently died and, as a consequence, the scheme was delivering:

‘150 to 200 hours less care [a week]. So for us now that is difficult, because I need to balance care needs, I need to balance budget, I also need to look at the kind of care levels that are coming in, cause clearly I need them to be quite high’ (SDM2).

She went on to describe the process of managing changes in the hours of care needs required by residents as a ‘constant battle’. Taken together these findings suggest that the capacity to respond flexibly to the changing care needs of residents, a central aspiration of ECH, was being compromised.

### 4.5 FAVOURS

Although there were formal processes through which to record and respond to unplanned care needs at each site, we also identified examples of care workers doing what we have called ‘favours’ for individual residents. ‘Favours’ were related to tasks that were not recorded in the care plan and did not appear in a ‘run’. While ‘favours’ were occasionally fitted into a shift, akin to what Dyck and England have termed ‘extras’ (2012), most often they were undertaken in the care workers’ own time. They related to activities that might better be thought of as informal or ‘familial’ care. For example, several care workers told us that, if asked, they would buy a resident a pint of milk if they were going shopping themselves, or they would post a letter for a resident on their way home. Doing these ‘favours’ appeared to operate as a strategy to compensate for the rigid way in which care was organised (Tufte, 2013). A care worker at site C, the specialist dementia setting, reported spending time decorating a cake with a male resident in his own apartment. She explained:

‘[…] it’s a bit of a task for him to do, just putting the sweets on but he seemed to enjoy it. Yeah so that was off my own back not something that was in the care plan’ (SCCW1).

Care workers often justified doing ‘favours’ on the basis that the resident did not have family members living locally. For example, at site D, a care worker told us that she occasionally did some laundry for a resident and painted another resident's nails ‘because I had a bit of time. It wasn't in the care plan but […] I sat with her and done that for her’ (SDCW5). Similarly, a care worker at site A described making alterations to an apron for a resident as well as visiting another resident in hospital. She said ‘I went to see her a couple of times. Took her washing, washed it, took it back’ (SACW2).

At each site, we were told by managers, and sometimes care workers, that they were not supposed to do such ‘favours’, usually citing reasons of efficacy and fairness. At site A, a team leader explained that it did not make any business sense to do unfunded work and it also led to a lack of equity between residents. She said:

‘[…] that’s not how the business should work and I strongly believe in that because it’s not about me being kind, but it’s not fair you know I’m kind of charging one person for the shopping but for the other one I will do for free […] where is the equality’ (SACW1).

## 5 DISCUSSION

Extra care housing was heralded by policy makers as a new model of housing with care for older people based on values of promoting independence and enabling older people to live in their own apartments and have more choice and control over their lives (DH, 2003). Many of the care workers and managers appreciated the different ethos that ECH was based on and found it to be a rewarding sector to work in. However, given the multiple pressures within the sector, it was not surprising that our data revealed their impact, particularly on the way in which care was organised and the challenges that this posed for managers and care workers. These pressures were intensified by changes in the population moving into ECH.

While ECH was originally designed as a model of housing with care that provides care in a manner which is flexible and responsive to the changing needs of residents, the multiple pressures faced by the schemes appear to have undermined this vision. A responsive and flexible approach to the provision of care and support necessitates funding levels that allow for some ‘slack’ in the staffing arrangements within ECH, for example the use of ‘floating support’ (Twyford, 2018). This would enable staff to respond to immediate changes in need without compromising the care provided to other residents or negatively impacting upon the working conditions and practices of care workers. Organising care into ‘runs’ is a pragmatic approach to staff management, one that replicates the organisation of care in traditional residential and domiciliary settings. However, organising care in this way undercuts ideas about flexibility and person‐centred care which are at the heart of the ECH ideal. The rigidity and timing of ‘runs’ appeared to undermine the sense of independence and control that ECH was meant to foster. For managers conscious of their budget, organising care into ‘runs’ is thought to be efficient of time and money. A further consequence of this practice, however, is that care work is reduced to a series of functional tasks, devoid of social interaction (Lee‐Treweek, 1997).

The financial and demographic pressures facing local authorities have, in some areas (including Area 1), resulted in changes to the eligibility criteria for publicly funded ECH, directly impacting the nature of care needs among ECH populations (Skills for Care, 2017; West, Shaw, Hagger, & Holland, 2017). The increasing prevalence of those living with dementia places additional demands on the organisation of care. In addition, the growing numbers of people moving into ECH with complex needs – such as alcohol dependency and/or behaviours that challenge – requires a flexible and person‐centred approach to care provision. Such changes to the ECH population are likely to result in more unplanned care needs, making care work less predictable and less amenable to current forms of workforce planning and scheduling.

The consequence of these changes is as yet undocumented, but this study reveals that the pressures in ECH mirror those faced by care workers in traditional settings. This is concerning given what we know about the impact which conditions of work in the social care sector have upon employee recruitment and retention. Elsewhere, low staffing is a common way to reduce costs, which results in care workers struggling to keep up with basic care activities, undermining their efforts to individualise caregiving and, in turn, causing stress, exhaustion and burnout (Lloyd, Banerjee, Harrington, Jacobsen, & Szebehely, 2014). While a ‘time‐and‐task’‐based approach to the delivery of care may reduce the costs of care provision in ECH (Garwood, 2010), for the care workers in this study, this approach restricted their ability to spend time with residents causing stress and frustration and, reducing their role to a series of tasks (Kadri et al., 2018). Indeed, the pressures that care workers face were similar to those encountered in the domiciliary care sector where the routinisation of work appears to have reduced the quality of care provided (Atkinson & Crozier, 2016). Such pressures may exacerbate the recruitment and retention of care staff in this sector (Moriarty, Manthorpe, & Harris, 2018).

The care workers in this study emphasised their alignment with the principles of ECH, valuing the relational aspects of care and the ability to encourage independence and choice among residents. Given the importance which care workers attached to the relational elements of their work, it is perhaps unsurprising that we found examples of them subverting some of the workplace pressures that they were experiencing by providing ‘favours’. In common with previous research, these ‘favours’ (or ‘extras’) were underpinned by person‐centred principles, such as talking to and interacting with residents, responding to their individual interests and needs (Dyck & England, 2012). ‘Favours’ were often targeted at those elements of care which are difficult to predict and measure; activities which are not easily subjected to a ‘time‐and‐task’ oriented systems of commissioning and workplace organisation but, viewed by care workers as essential to ‘meaningful care work’ (Tufte, 2013:110).

## 6 LIMITATIONS

This paper report findings drawn from a larger study explore the changing care needs of residents of ECH. While this paper provides some insight to the experiences of care workers it suggests the need for a more complex study incorporating other methods, such as diaries, as a means to determine the impact of the challenges faced by care workers on their working practices, as well as on the care received by residents.

## 7 CONCLUSION

As previous research has noted, ECH has the potential to support older people to live independently with care provided flexibly when required (Holland et al., 2015). However, the provision of extra care is not without its challenges and in the context of financial and demographic changes, the ability of care workers to respond flexibly, which was the founding premise of ECH, has become increasingly constrained. Far from offering an alternative to traditional forms of care, this article suggests that managerial concerns, driven by commissioning systems focused on ‘task’ rather than ‘experience’, are undermining attempts to provide person‐centred care. With this in mind, it is imperative that local authority commissioners work in partnership with providers to ensure that the founding principles of ECH are upheld. In addition, it is important to continue to research the experiences and perspectives of those working in ECH if we are to defend and preserve the important contribution that ECH can make in preserving the independence of people with increasing care needs.

## CONFLICT OF INTEREST

This article presents independent research funded by the NIHR School for Social Care Research. The views expressed in this publication are those of the authors and not necessarily those of the NIHR School for Social Care Research or the Department of Health and Social Care, NIHR or NHS.
